# Uptake of PSMA-ligands in normal tissues is dependent on tumor load in patients with prostate cancer

**DOI:** 10.18632/oncotarget.19049

**Published:** 2017-07-06

**Authors:** Florian C. Gaertner, Khalil Halabi, Hojjat Ahmadzadehfar, Stefan Kürpig, Elisabeth Eppard, Charalambos Kotsikopoulos, Nikolaos Liakos, Ralph A. Bundschuh, Holger Strunk, Markus Essler

**Affiliations:** ^1^ Department of Nuclear Medicine, University Hospital Bonn, Bonn, Germany; ^2^ Department of Radiology, University Hospital Bonn, Bonn, Germany

**Keywords:** PSMA, PET/CT, radionuclide therapy, prostate cancer, tumor load

## Abstract

Radioligand therapy (RLT) with Lu-177-labeled PSMA-ligands is a new therapy option for prostate cancer. Biodistribution in normal tissues is of interest for therapy planning. We evaluated if the biodistribution of Ga-68-PSMA-11 is influenced by tumor load.

**Results:**

In patients with high tumor load, SUV_mean_ was reduced to 61.5% in the lacrimal glands, to 56.6% in the parotid glands, to 63.7% in the submandibular glands, to 61.3% in the sublingual glands and to 55.4% in the kidneys (*p* < 0.001). Further significant differences were observed for brain, mediastinum, liver, spleen and muscle. Total tracer retention was higher in patients with high tumor load (*p* < 0.05). SUV in lacrimal, salivary glands and kidneys correlated negatively with PSA.

**Materials and Methods:**

135 patients were retrospectively evaluated. SUV was measured in the lacrimal and salivary glands, brain, heart, liver, spleen, kidneys, muscle and bone. SUV was correlated with visual tumor load, total tracer retention and PSA.

**Conclusions:**

Patients with high tumor load show a significant reduction of tracer uptake in dose-limiting organs. As similar effects might occur when performing RLT using Lu-177-labeled PSMA-ligands, individual adaptations of therapy protocols based on diagnostic PSMA PET imaging before therapy might help to further increase efficacy and safety of RLT.

## INTRODUCTION

The prostate-specific membrane antigen (PSMA) is a promising target for prostate cancer diagnostics and therapy. Chelator-coupled PSMA-ligands based on the Glu-urea-Lys motif suitable for nuclear medicine applications have been developed, e.g. PSMA-11 and PSMA-617 [1–4]. Due to the frequent high expression of PSMA on the surface of prostate cancer cells, which correlates with androgen independence, metastasis and disease progression [5], these compounds rapidly accumulate in prostate cancer lesions. Labeling with the positron-emitter Ga-68 facilitates PET imaging for detection of prostate cancer lesions [6] and labeling with the beta-emitting radionuclide Lu-177 enables PSMA-targeted radionuclide therapy. First results with Lu-177-labeled PSMA-617 already indicate good response rates with low short-term side-effects in patients with advanced, castrate-resistant, metastasized prostate cancer [7–13].

Accumulation of PSMA-ligands is also observed in non-tumorous normal tissues, such as liver, spleen, kidneys and salivary glands. Especially the salivary glands and the kidneys are regarded as dose limiting organs when performing PSMA-targeted radionuclide therapy [12]. The total tumor mass might affect uptake in dose-limiting normal organs due to a tumor steal effect. It is currently unknown if a tumor steal effect is applicable to PSMA-targeting radiotracers and to what extent it affects the biodistribution of the radiopharmaceutical in normal tissues. However, knowledge of these effects is fundamental for estimation of maximum tolerated activities and optimization of clinical radionuclide therapy protocols.

In the current study, we retrospectively analyzed uptake of the radiolabeled PSMA-ligand Ga-68-PSMA-11 in normal tissues in relation to tumor load in patients who underwent PET/CT for staging of prostate cancer.

## RESULTS

### Patient characteristics

A total of 135 patients undergoing Ga-68-PSMA-11 PET/CT were retrospectively evaluated. Primary indications for referral were staging of prostate cancer and evaluation for Lu-177-PSMA-617 therapy. Mean patient age was 71.8 ± 7.4 years (range 42–94 years), mean administered activity was 158 ± 30 MBq (range 105–261 MBq). Patients with low administered activities (< 100 MBq) were excluded from analysis. Mean incubation time was 89 ± 22 minutes (range 48–143 minutes). Mean PSA was 188.0 ± 412 ng/ml (range 0.02–2860 ng/ml). There were no adverse or clinically detectable pharmacologic effects in any of the 135 subjects.

As the diagnostic PET/CT scans did not include the area above the skull base, the parotid glands, lacrimal glands and brain were not in the field of view in 16 patients. Due to extensive bone metastases, normal bone SUV could not be evaluated in 37 patients. 2 patients had prior splenectomy preventing spleen SUV measurement. In 2 patients, only one kidney was evaluated due to functional impairment of the contralateral kidney. In 12 patients, the sublingual glands could not be delineated due to low uptake. In 8 patients one or both submandibular glands could not be delineated due to low uptake.

### Tumor load

**Figure 1:**
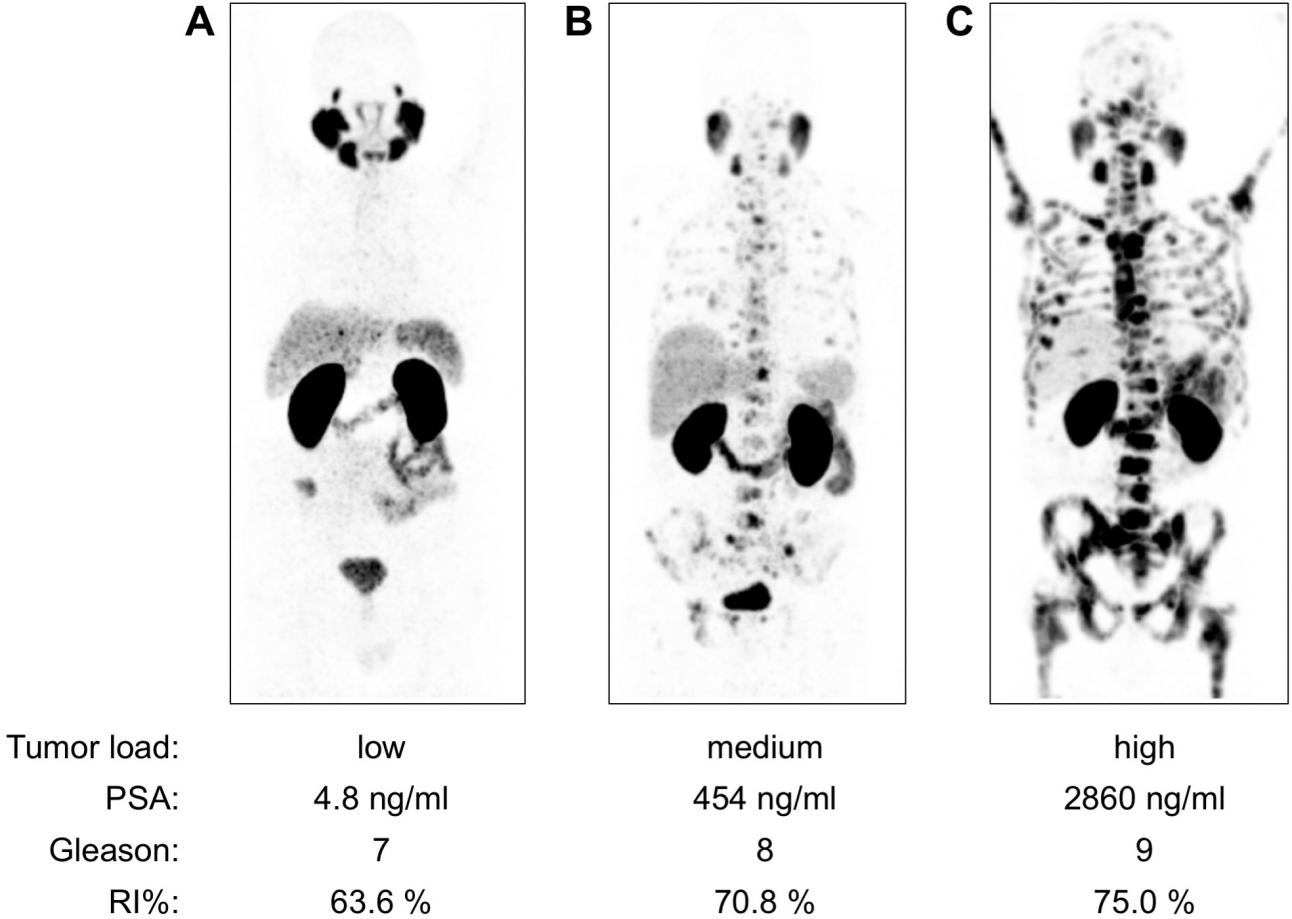
Examples of Ga-68-PSMA-11 PET/CT examinations (MIP projections, SUV 0–12) in patients with low, medium and high tumor load In patient (**A**) visually rated as low tumor load, PET/CT revealed two PSMA-positive bone metastases in the pelvis. Physiologic high uptake can be observed in the lacrimal glands, salivary glands (parotid, submandibular and sublingual glands) and in the kidneys. In patient (**B**) visually rated as medium tumor load, disseminated bone metastases with mostly faint to moderate intensity of tracer accumulation were observed, as well as multiple PSMA-positive lymph node metastases in the pelvis, abdomen and thorax. In patient (**C**) visually rated as high tumor load, intense Ga-68-PSMA-11 accumulation was present in the disseminated bone metastasis. Additionally, a small PSMA-positive local recurrence and small, Ga-68-PSMA-11-accumulating mediastinal lymph nodes were present. RI = retention index.

82 patients were rated as low tumor load, 19 as medium tumor load and 34 as high tumor load. Examples are shown in Figure 1.

**Figure 2:**
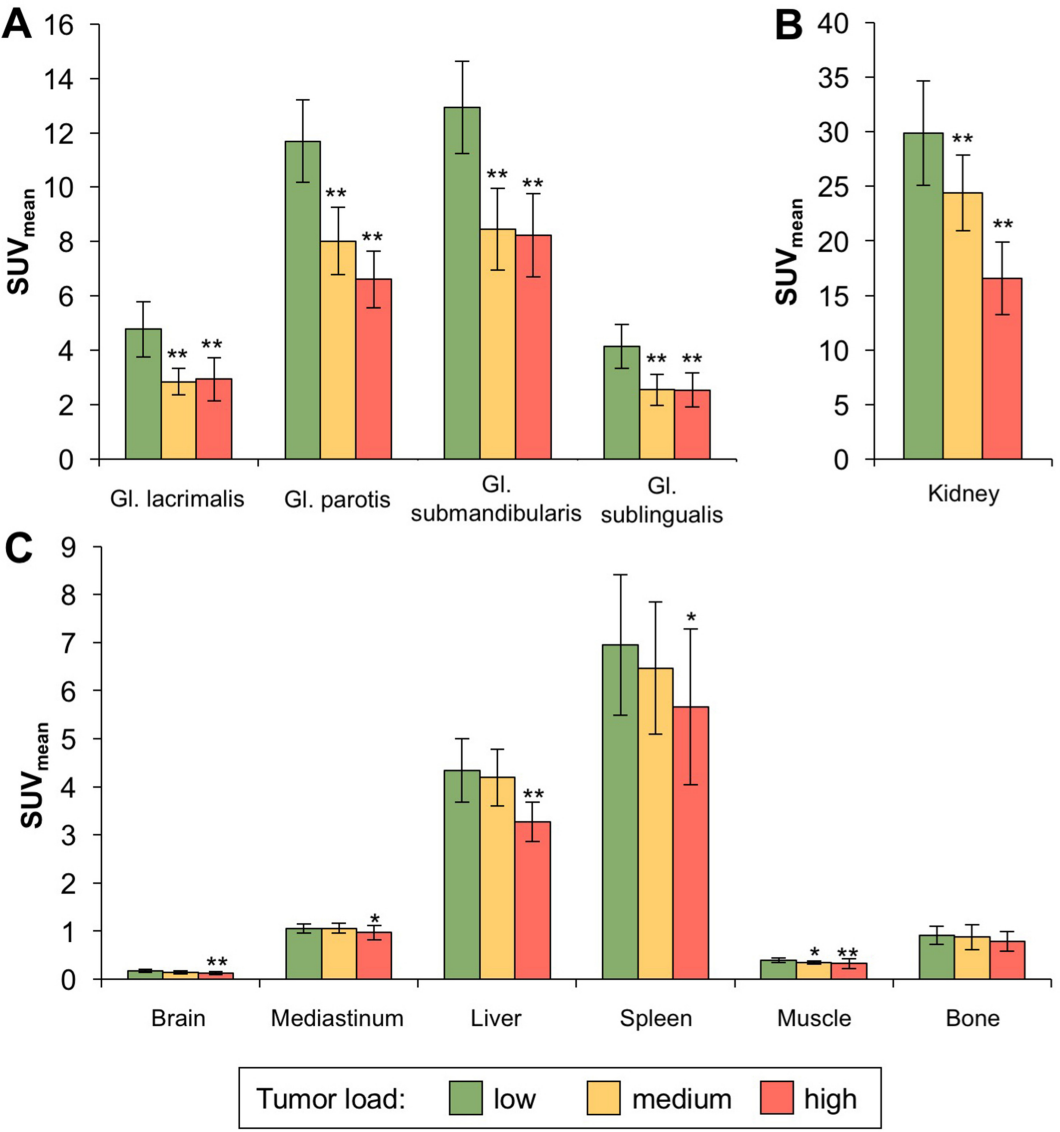
Reduction of Ga-68-PSMA-11 uptake in normal organs dependent on tumor load Bars represent mean uptake of Ga-68-PSMA-11 (SUV_mean_), error bars represent ± ½ SD. (**A**) In the lacrimal and salivary glands, a highly significant reduction in tracer uptake was observed in the groups of patients with medium and high tumor load. (**B**) Also in the kidneys, tracer uptake was markedly decreased in patients with medium and high tumor load with high significance. (**C**) Regarding the remaining normal tissues evaluated, patients with high tumor load showed significantly reduced tracer uptake in the brain, mediastinal blood pool, liver, spleen and muscle, however the amount of uptake reduction was less pronounced compared to the lacrimal/salivary glands and the kidneys. No significant differences were observed for bone uptake. *marks significant differences to the low tumor load group (*p* < 0.05). **marks highly significant differences to the low tumor load group (*p* < 0.001).

Tracer uptake in the lacrimal and salivary glands was highly dependent on tumor load, showing a significant reduction in patients with high tumor burden to values between 58% and 64% (*p* < 0.001), a graphical representation is shown in Figure 2A and detailed results are listed in Table 1. Tracer uptake in the kidneys was reduced to 58% (SUV_max_) and 55% (SUV_mean_), see Figure 2B.

**Table 1:** Accumulation of Ga-68-PSMA-11 in normal tissues dependent on visual analysis of tumor load

	Tumor load	*N*	SUV_max_ ± SD	% of low	*p* to low	SUV_mean_ ± SD	% of low	*p* to low
Lacrimal glands	Low	136	7.55 ± 3.32	-	**-**	4.77 ± 2.02	-	**-**
	Medium	34	4.76 ± 1.46	63.1%	**< 0.001**	2.85 ± 0.99	59.7%	**< 0.001**
	High	67	4.81 ± 2.47	63.7%	**< 0.001**	2.94 ± 1.57	61.5%	**< 0.001**
Parotid glands	Low	146	18.24 ± 4.60	-	**-**	11.69 ± 3.03	-	**-**
	Medium	36	13.00 ± 4.00	71.3%	**< 0.001**	8.02 ± 2.47	68.6%	**< 0.001**
	High	67	10.60 ± 3.41	58.1%	**< 0.001**	6.62 ± 2.09	56.6%	**< 0.001**
Sub-mandibular glands	Low	161	20.29 ± 5.21	-	**-**	12.93 ± 3.40	-	**-**
	Medium	36	13.92 ± 4.89	68.6%	**< 0.001**	8.46 ± 2.99	65.4%	**< 0.001**
	High	62	13.06 ± 4.73	64.4%	**< 0.001**	8.24 ± 3.06	63.7%	**< 0.001**
Sublingual glands	Low	80	7.05 ± 3.12	-	**-**	4.15 ± 1.62	-	**-**
	Medium	15	4.36 ± 1.86	61.9%	**< 0.05**	2.55 ± 1.12	61.5%	**< 0.001**
	High	28	4.22 ± 2.39	59.8%	**< 0.001**	2.54 ± 1.24	61.3%	**< 0.001**
Kidneys	Low	162	50.2 ± 15.55	-	**-**	29.9 ± 9.59	-	**-**
	Medium	38	43.1 ± 10.27	85.9%	**< 0.05**	24.4 ± 6.91	81.6%	**< 0.001**
	High	68	29.2 ± 11.10	58.3%	**< 0.001**	16.6 ± 6.72	55.4%	**< 0.001**
Spleen	Low	82	9.97 ± 3.53	-	**-**	6.95 ± 2.92	-	**-**
	Medium	18	9.26 ± 3.28	92.9%	> 0.05	6.47 ± 2.76	93.1%	> 0.05
	High	33	8.28 ± 4.03	83.1%	**< 0.05**	5.66 ± 3.23	81.4%	**< 0.05**
Liver	Low	81	7.58 ± 1.91	-	**-**	4.34 ± 1.33	-	**-**
	Medium	19	7.26 ± 1.75	95.8%	> 0.05	4.19 ± 1.19	96.6%	> 0.05
	High	32	6.19 ± 1.50	81.7%	**< 0.05**	3.27 ± 0.81	75.4%	**< 0.001**
Mediastinum	Low	82	2.99 ± 0.61	-	**-**	1.06 ± 0.19	-	**-**
	Medium	19	3.25 ± 0.82	108.7%	> 0.05	1.06 ± 0.21	100.4%	> 0.05
	High	34	2.87 ± 1.07	96.0%	> 0.05	0.97 ± 0.29	91.9%	**< 0.05**
Bone	Low	163	2.50 ± 0.92	-	**-**	0.91 ± 0.39	-	**-**
	Medium	11	2.67 ± 1.73	106.9%	> 0.05	0.88 ± 0.52	96.0%	> 0.05
	High	10	2.75 ± 1.47	110.2%	> 0.05	0.79 ± 0.41	86.0%	> 0.05
Muscle	Low	82	1.27 ± 0.28	-	**-**	0.39 ± 0.09	-	**-**
	Medium	18	1.30 ± 0.35	102.3%	> 0.05	0.35 ± 0.07	89.5%	**< 0.05**
	High	34	1.32 ± 0.81	103.7%	> 0.05	0.33 ± 0.20	82.7%	**< 0.001**
Brain	Low	68	0.78 ± 0.26	-	**-**	0.18 ± 0.07	-	**-**
	Medium	17	0.66 ± 0.23	84.5%	> 0.05	0.14 ± 0.06	80.7%	> 0.05
	High	34	0.64 ± 0.26	82.7%	**< 0.05**	0.12 ± 0.06	70.0%	**< 0.001**

Legend: The Wilcoxon-Mann-Whitney test was used to test for significance of uptake reduction in patients with medium and high tumor load compared to patients with low tumor load. Significant *p*-values (< 0.05) are highlighted by bold face type.

The effects of tumor load on tracer uptake in other normal organs was less pronounced, in the spleen tracer uptake was reduced to values from 83% to 81%, in the liver from 82% to 75% and in the brain from 83% to 70% (*p* < 0.05). SUV_mean_ in the mediastinum and muscle showed only a slight, but significant reduction to 92% and 83%, respectively (*p* < 0.05). No significant differences were observed for uptake in normal bone (Figure 2C, Table 1).

### Retention index (RI%)

Patients categorized as low tumor load (*n* = 82) showed a total tracer retention of 70.7% ± 7.4%. The results of patients with medium tumor load were not significantly different (71.0% ± 8.8%, *p* > 0.05, *n* = 19). A slightly higher tracer retention was observed in patients with high tumor load (74.0% ± 7.6%, *p* < 0.05, *n* = 34).

There was a slight, but significant negative correlation of RI% and uptake in the lacrimal, parotid and submandibular glands. No significant correlation was observed for sublingual gland and kidney uptake (Table 2).

**Table 2:** Correlation of Ga-68-PSMA-11 uptake with retention index and serum PSA

	Correlation of RI% with SUV			Correlation of PSA with SUV		
		SUV_max_	SUV_mean_		SUV_max_	SUV_mean_
	*N*	rho (*p*^a^)	rho (*p*^a^)	*N*	rho (*p*^a^)	rho (*p*^a^)
Lacrimal glands	237	−0.277 (< 0.001^b^)	−0.263 (< 0.001^b^)	235	–0.346 (< 0.001^b^)	–0.398 (< 0.001^b^)
Parotid glands	249	−0.169 (< 0.05^b^)	−0.158 (< 0.05^b^)	247	–0.551 (< 0.001^b^)	–0.569 (< 0.001^b^)
Submandibular glands	259	−0.129 (< 0.05^b^)	−0.144 (< 0.05^b^)	257	–0.412 (< 0.001^b^)	–0.448 (< 0.001^b^)
Sublingual glands	123	0.000 (> 0.1)	−0.048 (> 0.1)	121	–0.376 (< 0.001^b^)	–0.374 (< 0.001^b^)
Kidneys	268	0.062 (> 0.1)	0.063 (> 0.1)	266	–0.487 (< 0.001^b^)	–0.517 (< 0.001^b^)

Legend: Correlation coefficients (Spearman's rho) of Ga-68-PSMA-11 uptake (SUV_max_ and SUV_mean_) with retention index (RI%, left side of the table) and serum PSA (right side of table) regarding the lacrimal and salivary glands and the kidneys. Low negative correlation coefficients were observed for correlation of SUV with RI% for the lacrimal, parotid and submandibular glands. More pronounced negative correlations were observed for correlation of SUV with serum PSA.

^a^two-tailed.

^b^marks significant correlations (*p* < 0.05).

### PSA

Serum PSA was available for 133 of 135 patients. Mean PSA in patients categorized as low tumor load (*n* = 80) was 25.3 ± 69.0 ng/ml. In patients with medium tumor load (*n* = 19), mean PSA was significantly higher (298 ± 299 ng/ml, *p* < 0.001). Also, patients with high tumor load (*n* = 34) showed a significantly higher PSA than patients with low tumor load (509 ± 663 ng/ml, *p* < 0.001).

**Figure 3:**
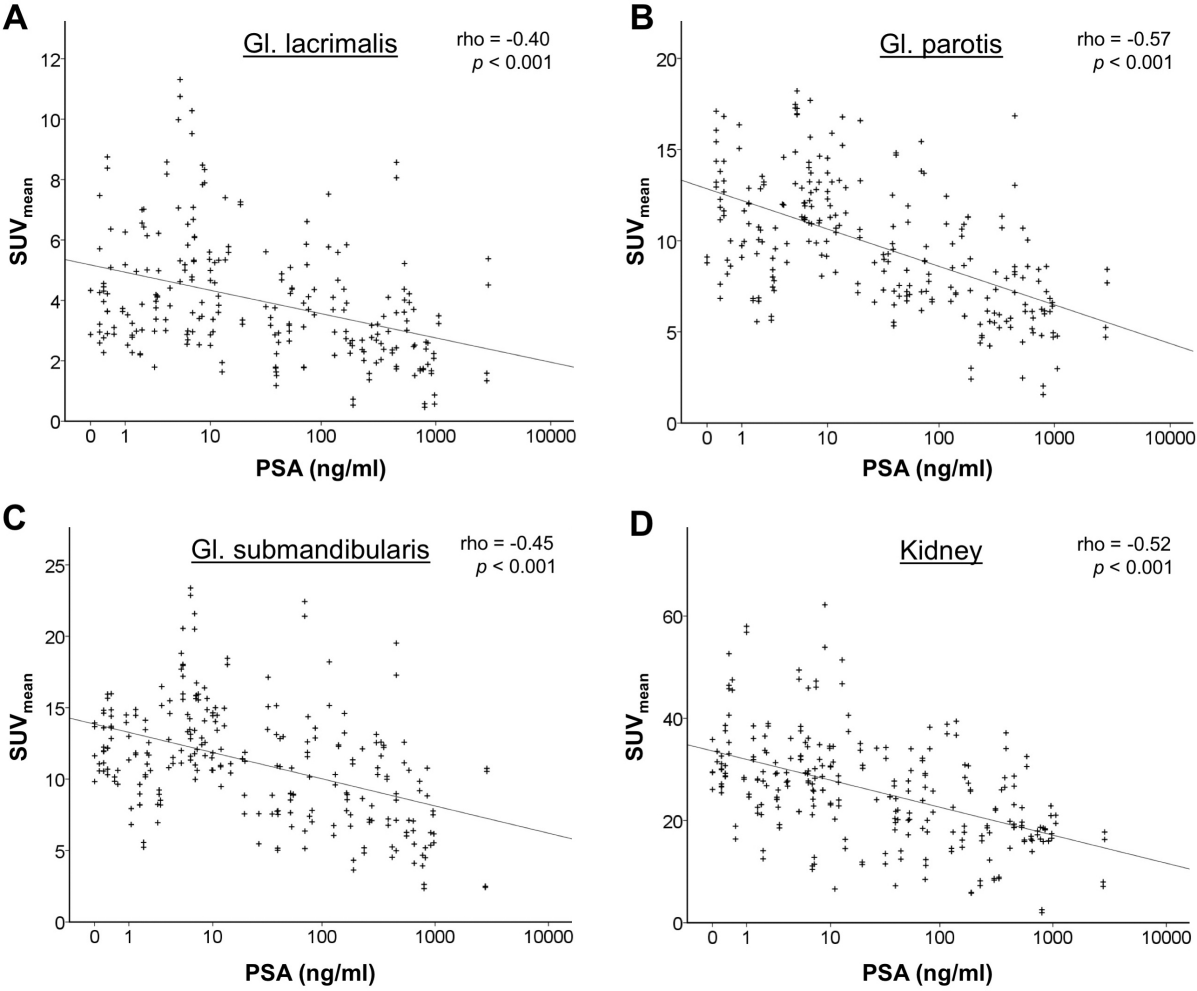
Correlation of Ga-68-PSMA-11 uptake in normal organs (SUV_mean_) with serum PSA as a surrogate of tumor load Highly significant (*p* < 0.001) negative correlations were observed for the lacrimal glands, salivary glands and the kidneys, however the correlation coefficients (Spearman's rho) were only in a moderate range, indicating a remaining high variability of tracer uptake.

There were significant negative correlations of serum PSA and uptake in the lacrimal and salivary glands, as well as in the kidneys, however the correlation coefficients were only in the moderate range, see Table 2 and Figure 3.

A significant (*p* < 0.05) reduction of mean SUV in the salivary glands and kidneys was observed at PSA thresholds of 6–9 ng/ml and above, and a reduction of more than one standard deviation was mostly observed at PSA thresholds above 500–900 ng/ml (see Table 3).

**Table 3:** PSA thresholds for significant reduction of Ga-68-PSMA-11 uptake

	SUV_max_		SUV_mean_	
	significant reduction if PSA	reduction > 1 SD if PSA	significant reduction if PSA	reduction > 1 SD if PSA
Lacrimal glands	> 9 ng/ml	N/A	> 8 ng/ml	N/A
Parotid glands	> 7 ng/ml	> 500 ng/ml	> 6 ng/ml	> 500 ng/ml
Submandibular glands	> 7 ng/ml	> 500 ng/ml	> 7 ng/ml	> 500 ng/ml
Sublingual glands	> 6 ng/ml	> 900 ng/ml	> 7 ng/ml	> 900 ng/ml
Kidneys	> 6 ng/ml	> 800 ng/ml	> 6 ng/ml	> 800 ng/ml

Legend: When calculating mean SUV of potential dose-limiting organs in patients with serum PSA above a certain threshold, a significant reduction (*p* < 0.05) in comparison to all patients was observed at PSA thresholds above 6–9 ng/ml for SUV_max_ and SUV_mean_. Reduction of mean SUV of more than one standard deviation was observed at PSA levels above 500–900 ng/ml.

### Incubation time and injected activity

Significant correlations (*p* < 0.05) between incubation time and uptake were only observed for SUV_mean_ with very low correlation coefficients in the mediastinum (rho = −0.209) and in the spleen (rho = –0.213). No significant correlations were observed for SUV_mean_ in all other organs tested and for SUV_max_ in all organs (*p* > 0.05).

Significant correlations (*p* < 0.05) between injected activity and uptake were observed with very low correlation coefficients for SUV_mean_ in the lacrimal glands (rho = 0.199), and for SUV_max_ in the lacrimal glands (rho = 0.231), in the kidneys (rho = 0.120) and in the bone (rho = 0.172). No significant correlations were observed for SUV_mean_ or SUV_max_ in all other organs tested (*p* > 0.05).

### Gleason score and age

Gleason score was available for 107 of 135 patients. Most patients referred for Ga-68-PSMA-11 PET/CT had a Gleason score between 6 and 9 (104 patients), with only 2 patients showing a Gleason score of 10 and one patient with an initial Gleason score of 4. There was a trend of patients with a higher Gleason score to present with a higher tumor load. In the group of patients with low tumor load, the median Gleason score was 7, and patients with medium and high tumor load had a median Gleason score of 8 and 9, respectively.

There was a tendency of a higher tumor load to be present with increasing age. The mean patient age in the group with low tumor load was 70.4 ± 7.3 years, whereas the values for medium and high tumor load were 73.4 ± 6.5 years and 74.3 ± 7.8 years, respectively. The age difference between the low and high tumor load groups was significantly different (*p* < 0.05).

## DISCUSSION

We observed a pronounced influence of tumor load on the biodistribution of radiolabeled PSMA-ligands in normal tissues. A mean reduction of activity concentration by almost 50% was observed in some organs in patients with high tumor load, in individual cases markedly exceeding 50% reduction. Especially, a high dependence of tracer uptake on tumor load was observed in the kidneys, as well as in the salivary glands and lacrimal glands. These organs exhibit high physiological uptake of radiolabeled PSMA-ligands and are therefore considered dose-limiting organs when performing radionuclide therapy using Lu-177-labeled PSMA-binding constructs [12].

For external beam radiotherapy of the kidneys, a tolerance dose of 23 Gy is widely accepted [14]. Regarding the salivary glands, Deasy et al. reported that severe xerostomia can usually be avoided if the absorbed dose in both parotid glands is limited to 25 Gy [15]. In a study with 7 patients undergoing therapy with Lu-177-PSMA-617, Kabasakal et al. estimated a mean absorbed dose in the parotid glands of 1.17 Gy / GBq and in the kidneys of 0.88 Gy/GBq [16]. They reported a high inter-patient variability for absorbed doses, which is in line with our current results that additionally point to a significant dependence of radiotracer uptake on tumor load. For the same compound, similar values were reported by Delker et al. with 1.4 Gy/GBq for the salivary glands and 0.6 Gy/GBq for the kidneys in 5 patients (2 cycles of therapy each) [17], and by Kratochwil et al. with 1.4 Gy/GBq for the salivary glands and 0.75 Gy/GBq for the kidneys [18]. Regarding another PSMA-ligand, Lu-177-DOTAGA-(I-y)fk (Sub-KuE), the parotid dose was reported as 1.3 Gy/GBq and the kidney dose was 0.8 Gy/GBq [19]. Regarding Lu-177-PSMA-I&T, the parotid dose was 0.55 Gy/GBq and the dose to the kidneys was reported as 0.72 Gy/GBq [20]. Extrapolating from these data, a mean absorbed dose of 23 Gy in the kidneys would be reached by a cumulative activity of 26–38 GBq Lu-177-PSMA-ligand. For the parotid glands, a mean absorbed dose of 25 Gy would be reached by the application of a cumulated activity of 18–45 GBq Lu-177-PSMA-ligand. These cumulative activities are in the range of currently discussed clinical therapy protocols for radionuclide therapy using Lu-177-labeled PSMA-ligands.

Our current results indicate that patients with high tumor load might tolerate significantly higher activity amounts of Lu-177-labeled PSMA-ligands before adverse effects on kidney and salivary gland function become relevant. However, the restriction to single static scans is a limitation of our study. A dosimetry study with several intra-individual time points is needed to confirm these results.

Kratochwil et al. performed a dosimetry study with Lu-177-PSMA-617 in 4 patients (2 cycles of therapy per patient), however they did not observe a dependency of salivary gland or kidney dose on tumor burden, which however might be attributable to the small number of patients evaluated [18].

Though a fraction of the patients in our collective subsequently underwent Lu-177-PSMA-617 therapy in the further clinical course, the number of patients is not sufficient to assess if a correlation exists between pre-therapeutic salivary gland uptake and the occurrence of post-therapeutic xerostomia. To evaluate this, a prospective clinical trial including a higher number of patients is needed.

Furthermore, even when taking tumor load into account, inter-patient variability of tracer uptake in the kidneys and salivary glands remains high. Thus, incorporating a patient's individual biodistribution data, not only based on visual evaluation of tumor load and/or serum PSA measurement, but additionally on kidney and salivary gland uptake in diagnostic PSMA PET imaging before therapy or even by performing pre-therapeutic dosimetry seems rational for individual therapy planning to further optimize the efficacy of PSMA-targeted radionuclide therapy. Individual adaptations of therapy protocols might result in increased number of therapy cycles and/or increased activity amount per cycle in patients with low kidney and salivary gland uptake. Also, variable amounts of activity per cycle might be discussed, with higher amounts being applied during the first cycles, to account for decreasing tumor load during the course of radionuclide therapy.

Patients with high tumor load showed a slightly, yet significantly higher tracer retention in the body trunk than patients with low tumor load. This corroborates the hypothesis that extensive tumor uptake leads to less radiotracer being available for uptake in background organs, reducing accumulation in these organs, in terms of a tumor steal effect. However, as the retention index is only slightly elevated in patients with high tumor load and the correlation with tracer uptake yields only low correlation coefficients, this parameter does not seem feasible for quantitation of tumor load.

It is a limitation of our study that a systematic error in calculation of the retention index is present as the PET acquisitions did not include the lower extremities, and in some cases where a diagnostic CT protocol was performed additionally excluded the neurocranium, thus there is a tendency for underestimation of the retention index. However, as potential tracer accumulation in the head and legs is minimal compared to the body trunk, the estimated error is low and in our opinion negligible. Nevertheless, as distant metastases of prostate cancer may occur as singular metastases of lower legs, whole body acquisition of PSMA-PET should be considered in clinical imaging protocols.

The nasal mucosa, which sometimes shows accumulation of PSMA-ligands, has not been included in our evaluation. However, per Hohberg et al., absorbed organ doses of Lu-177-PSMA-therapy are not likely to be critical for the nasal mucous membrane, as typical therapy protocols result in a dose of about 7 Gy to the nasal mucosa, whereas the commonly applied dose constraint to this organ is 37 Gy [21].

Another limitation of our study are the variations in injected activity and incubation time, which might influence SUV measurements. Significant correlations of uptake with incubation time were observed for SUV_mean_ in the mediastinum and in the spleen with very low correlation coefficients (rho ≈ −0.2). When testing for correlations between injected activity and uptake, significant correlations were observed in the lacrimal glands, kidneys and in the bone, but again with only very low correlation coefficients (rho ≈ 0.1 to 0.2). To exclude a relevant influence of these parameters on the result of this study, we tested for dependencies of tumor load (PSA) with incubation time and injected activity. There were no significant correlations between tumor load and incubation time (*p* > 0.05) or between tumor load and injected activity (*p* > 0.05). Therefore, due to the low correlation coefficients, as well as to the missing dependencies of incubation time and injected activity on tumor load, a relevant influence of these parameters on the general result of this study can be excluded.

A significant reduction of mean tracer uptake in the salivary glands and kidneys was observed already at PSA levels above 6–9 ng/ml, and pronounced mean reductions in tracer accumulation of more than one standard deviation were observed when PSA was above 500–900 ng/ml. These calculations furthermore illustrate the reciprocal dependency of salivary gland and kidney uptake on tumor load. Nevertheless, even when taking tumor load and/or serum PSA into account, a pronounced variability of salivary gland and kidney uptake remains evident. Therefore, these values should not be regarded as cut-off thresholds for clinical decision making. Instead, the patient's individual biodistribution in the target organs derived from diagnostic PSMA PET imaging before therapy appears to provide a more reliable basis for individual planning of PSMA-targeted radionuclide therapy.

## MATERIALS AND METHODS

### PET imaging

Glu-NH-CO-NH-Lys-(Ahx)-[Ga-68-HBED-CC] (PSMA-ligand, in the following referred to as Ga-68-PSMA-11) was produced using ethanol post-processed Ga-68 eluate [22], 1000 μl 1 M ammonium acetate buffer and 3–5 nmol PSMA-11. The reaction mixture was heated to 85°C for 5 min (pH 3.9–4.2), diluted with saline and sterile filtrated. Precursor was from ABX (Radeberg, Germany). Ge-68/Ga-68 generator was from iThemba Labs (Cape Town, South Africa). Overall synthesis yield was > 90%, radiochemical purity was > 99%. Specific activity was 0.24–0.4 GBq/nmol.

Patients were injected i.v. with 20 mg furosemide, followed by Ga-68-PSMA-11 (2 MBq/kg body weight). The average dose was 158 ± 30 MBq (range 105–261 MBq). PET/CT was performed on a Biograph 2 PET/CT scanner (Siemens Medical Solutions, Erlangen, Germany). PET emission data was acquired in 3D mode, emission time was 4 minutes per bed position. PET images were reconstructed iteratively (attenuation weighted OSEM, 4 iterations, 8 subsets), including scatter, random and decay correction. Images were smoothed by a 5 mm Gaussian filter. Attenuation correction was performed using CT data. Depending on the clinical situation, either a diagnostic CT (80 mAs, 130 kV, DoseCare4D) including the application of i.v. and oral contrast or a low-dose CT (16 mAs, 130 kV) without i.v. contrast was performed.

The study has been approved by the institutional review board. For this type of study (retrospective analysis) the need for written informed consent was waived. All procedures performed were in accordance with the ethical standards of the institutional and/or national research committee and with the principles of the 1964 Declaration of Helsinki and its later amendments or comparable ethical standards.

### Image analysis

Images were analyzed using Interview Fusion software (Version 3.00.060.0000, Mediso Ltd., Budapest, Hungary). Volumes of interest (VOIs) were drawn as follows: 50% isocontour for lacrimal glands, parotid glands, submandibular glands, sublingual glands and kidneys. As the sublingual glands sometimes only showed faint tracer uptake, the isocontour threshold was increased to visually match the sublingual gland outline to prevent oversegmentation in these cases. Spherical VOIs were used for the following organs: 50 mm diameter for brain, mediastinum, liver and muscle (right gluteal region); 30 mm diameter for spleen; 25 mm diameter for sacral bone and lumbar spine bone. Care was taken not to include metastases and to encompass a region of homogeneous tracer uptake in the PET dataset as well as to confine to morphologic organ bounds using the CT dataset. Regarding lumbar spine, measurement of L3 was preferred. If L3 contained a metastasis, L4 and subsequently L5 was used. SUV_max_ and SUV_mean_ (based on body weight, including correction for physical decay of Ga-68) were calculated for all VOIs.

The retention index (RI%) was calculated by placing a rectangular VOI over the whole PET data set to measure total activity in the body trunk (MBq). Urinary bladder contents were delineated visually to determine total bladder activity (MBq), which was then subtracted from the total body trunk activity. The resulting difference, representing total activity remaining in body tissues at the time of PET imaging (MBq) was corrected for physical decay to the time point of tracer injection and additionally normalized to the injected activity. Therefore, RI% represents the percentage of radiotracer remaining in the body after the incubation period.

Tumor load was analyzed visually to categorize the patients into three groups: low, medium and high tumor load. Patients with disseminated bone metastases (> 30 single lesions or diffusely confluent bone lesions encompassing more than half of the skeleton) showing intense uptake were defined as high tumor load. Patients with disseminated bone metastases showing only faint uptake or encompassing less than half of the skeleton, extensive PSMA-ligand accumulating lymph node bulks on both sides of the mediastinum or extensive PSMA-positive lung metastases were classified as medium tumor load. All other patients were classified as low tumor load.

### Statistics

The Wilcoxon-Mann-Whitney test was used to test for reduction of tracer uptake in the patient groups based on tumor load. *p* < 0.05 was rated as significant and *p* < 0.001 as highly significant. Correlation of SUV with PSA and RI% were evaluated by calculating the correlation coefficient (Spearman's rho) with a two-tailed test for significance.

## Abbreviations

MIP: maximum intensity projection
OSEM: ordered subset expectation maximization
PET: positron emission tomography
PSA: prostate specific antigen
PSMA: prostate specific membrane antigen
RI%: retention index
SD: standard deviation
SUV: standardized uptake value
VOI: volume of interest
